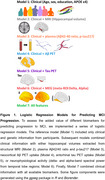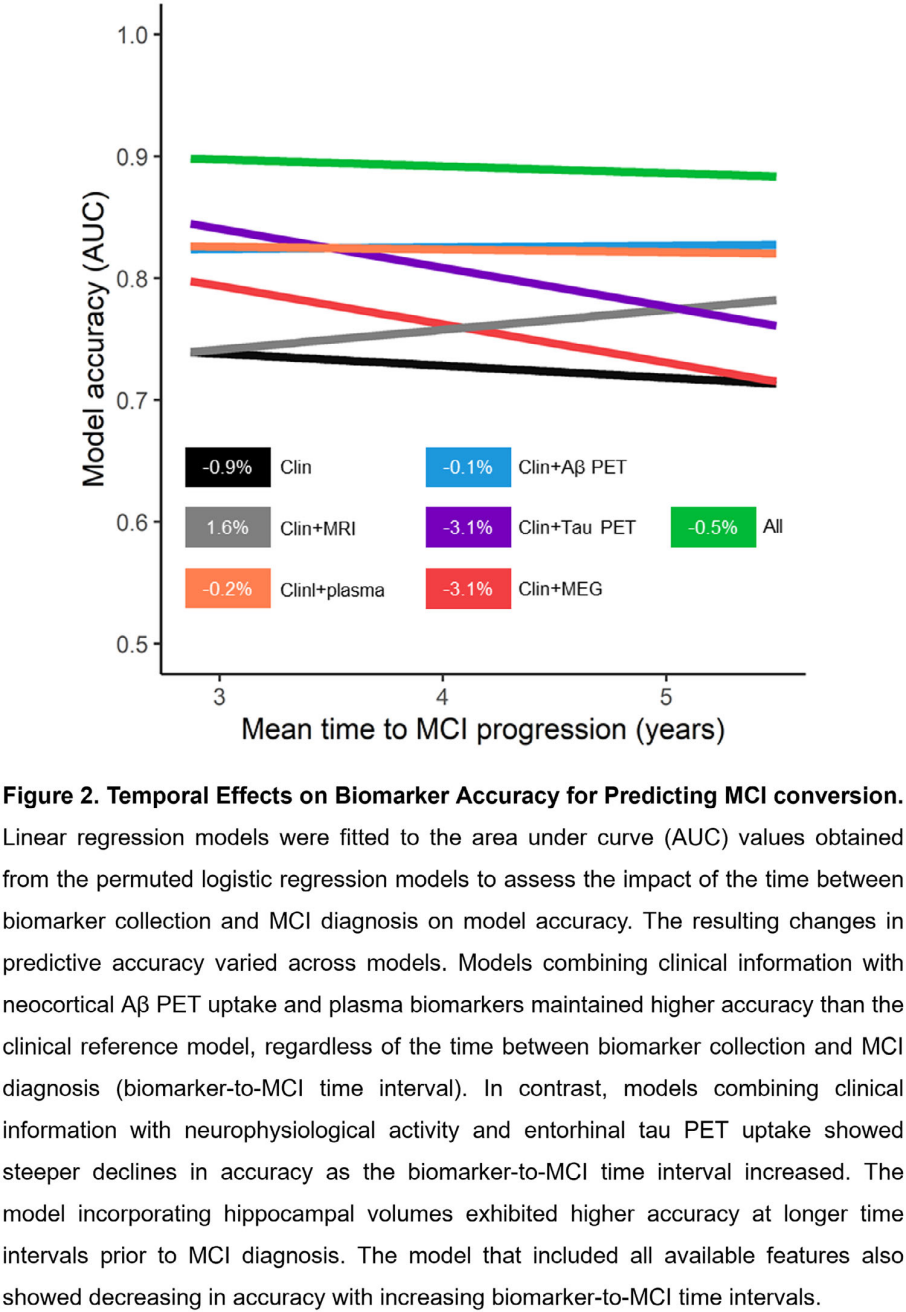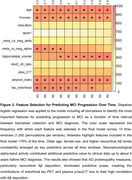# Time‐dependent biomarker accuracy in forecasting mild cognitive impairment

**DOI:** 10.1002/alz70856_101184

**Published:** 2025-12-25

**Authors:** Jonathan Gallego Rudolf, Alex I Wiesman, Sylvain Baillet, Sylvia Villeneuve, PREVENT‐AD Research group

**Affiliations:** ^1^ Douglas Research Centre, McGill University, Montreal, QC, Canada; ^2^ Montreal Neurological Institute, McGill University, Montreal, QC, Canada; ^3^ Department of Neurology and Neurosurgery, McGill University, Montreal, QC, Canada; ^4^ Department of Psychiatry, McGill University, Montréal, QC, Canada

## Abstract

**Background:**

Several biomarkers have been proposed for predicting the risk of progression of asymptomatic individuals to mild cognitive impairment (MCI). However, there is the need of characterizing the dynamic change in their accuracy as a function of the time between biomarker collection and MCI diagnosis. In addition, the potential contribution of direct measures of neurophysiological activity for estimating the risk of MCI progression has not been explored in depth.

**Method:**

We assessed spectral power features from task‐free magnetoencephalographic (MEG) recordings, MRI‐derived hippocampal volumes, plasma biomarkers, and PET measures of Aβ and tau deposition in a group of cognitively unimpaired older adults with a family history of AD (*N* = 102). From this sample, 31 individuals developed MCI based on a multidisciplinary consensus who had access to longitudinal neuropsychological assessments but were blind to biomarker information (mean time between biomarkers collection and MCI diagnosis = 4 years; SD = 1.9 years). We benchmarked these biomarkers using a series of logistic regression models to assess the temporal evolution of their accuracy for predicting MCI progression, in combination with clinical information (Figure 1).

**Result:**

Neurophysiological activity features and tau pet provided additional information to the clinical model when acquired up to ∼4 years prior to diagnosis, but their accuracy decreased at larger intervals. In contrast, the accuracy gained by incorporating Aβ PET or plasma biomarkers remained high up to 6 years before diagnosis (Figure 2). These observations were confirmed after running stepwise logistic regression on the model including all biomarkers, highlighting the contribution of age, sex, plasma *p*‐tau217, Aβ and tau PET and neurophysiological activity for predicting MCI progression at different time intervals (Figure 3).

**Conclusion:**

Overall, our results delineate a timeline of the accuracy provided by different biomarkers for predicting progression to MCI. Such findings highlight the dynamic sensitivity of different biomarkers, which is dependent on the time lapse between biomarkers collection and clinical diagnosis. This is particularly relevant for future clinical trials that intend to use biomarkers for screening participants.